# Redtail and red colobus monkeys show intersite urinary cortisol concentration variation in Kibale National Park, Uganda

**DOI:** 10.1093/conphys/cov006

**Published:** 2015-03-13

**Authors:** Gary P. Aronsen, Melanie M. Beuerlein, David P. Watts, Richard G. Bribiescas

**Affiliations:** Department of Anthropology, Yale University, New Haven, CT, USA

**Keywords:** Ecophysiology, fragmentation, Kibale, predation, primates, stress

## Abstract

Non-invasive measurement of urinary cortisol is a proven method of evaluating the impact of environmental stressors on wild primates. Variation in cortisol concentrations can reflect physiological stress, and prolonged elevation of circulating cortisol can significantly affect individual and population-level health. In a previous study, we found that urinary cortisol concentrations in grey-cheeked mangabeys (*Lophocebus albigena*) were higher at a highly disturbed site (Mainaro) in Kibale National Park, Uganda compared with a minimally disturbed site (Ngogo) in the same habitat. Here, we expand on this research, reporting on cortisol concentrations in two other cercopithecid monkeys (*Cercopithecus ascanius* and *Piliocolobus rufomitratus*) at the same two sites. We hypothesized that *C. ascanius* would show no difference between sites, given its preference for secondary forests, while *P. rufomitratus* would have higher cortisol concentrations at the disturbed site. Contrary to expectations, both species exhibited significantly higher cortisol concentrations at Ngogo (minimally disturbed) compared with Mainaro (disturbed). We suggest that these results may be caused by inter- or intragroup social dynamics, intersite differences in predation rate, fruit/food availability and chemistry, or a combination of these factors. These initial evaluations of urinary cortisol concentrations provide testable hypotheses on habitat disturbance and Kibale primate ecophysiology.

## Abstract (precis)

We compare cortisol levels in monkeys at two sites with varying habitat disturbance within Kibale National Park, Uganda. Both species have higher cortisol levels at the less disturbed of the two sites. Factors such as social dynamics or predation may be responsible, illustrating the subtleties of wild primate ecophysiology.

## Introduction

Deterioration of environmental quality due to factors such as drought, increased predation and habitat disturbance can affect wild animal population health. Social factors, including competition for food and mating opportunities and instability in social relationships, can also influence well-being. Ecological or social perturbations may disrupt homeostasis and force immediate physiological adjustments, changing the hormonal milieu that regulates growth, immune function and reproduction (Reeder and Kramer, 2005). The development of methods to analyse stress biomarkers via non-invasive collection of urine and faeces from wild populations provides insights into ecophysiology in general (Wikelski and Cooke, 2006; Romano *et al.*, 2010; Cooke *et al.*, 2013b). Primate habitat disturbance varies greatly along a spectrum from large, relatively undisturbed swathes of suitable habitat (e.g. closed canopy forests) minimally affected by human activity to small, threatened fragments in landscapes extensively altered by humans (Johns and Skorupa, 1987; Bicca-Marques, 2003; Marsh, 2003). Comparing primate communities in undisturbed vs. disturbed habitats can illustrate the influence of environmental conditions on physiological stress.

Cortisol is a glucocorticoid produced by the adrenal cortex under stimulation by adrenocorticotrophic hormone. It is responsible for classic stress responses to environmental challenges. It acts as an anti-inflammatory agent, facilitates the cellular uptake of glucose and acts as a catabolic agent. Its molecular structure and function are conserved across most vertebrates and all mammals, making it a viable and robust agent of investigation for conservation biology (Griffin and Ojeda, 2004; Norris and Carr, 2013). Multiple stressors can stimulate increases in cortisol, such as food shortages, social instability, infection and injury, predation threat and habitat disturbance (Sapolsky, 2005; Honess and Marin, 2006; Wikelski and Cooke, 2006; Anestis, 2010). While the functional utility of short-term increases in cortisol are well known, chronically elevated cortisol can disrupt reproductive function, immunocompetence, growth and neurological function (Sapolsky *et al*., 1990; Chrousos *et al*., 1998; Habib *et al*., 2000; Boonstra, 2013a,b). Cortisol concentrations in primate urine reflect the amount of the hormone circulating in both conjugated and unconjugated forms (Bahr *et al*., 2000) and can be assessed using samples collected non-invasively in the field (Whitten *et al*., 1998). As analytical methods improve, primatologists are providing more nuanced analyses of wild primate physiology and habitat-associated stressors such as habitat disturbance (Cavigelli, 1999; Muller and Wrangham, 2004; Strier and Ziegler, 2005; Chapman *et al*., 2006; Martínez-Mota *et al*., 2007; Dittami *et al*., 2008; Arlet *et al*., 2009; Arlet and Isbell, 2009; Shutt *et al*., 2012; Rimbach *et al*., 2013; Balestri *et al*., 2014).

We previously found that urinary cortisol concentrations were higher in grey-cheeked mangabeys (*Lophocebus albigena johnstoni*) at a highly disturbed site than in conspecifics at a relatively undisturbed site in Kibale National Park, Uganda, presumably due to the physiological stress of living in the disturbed habitat (Jaimez *et al*., 2012). Here, we expand on this research to determine whether two other cercopithecid primates (redtail monkeys, *Cercopithecus ascanius schmidti*, and red colobus monkeys, *Piliocolobus rufomitratus tephrosceles*), sympatric with mangabeys at both sites, show similar disturbance-related differences in cortisol concentrations. We predicted that red colobus monkeys would show such differences because, like mangabeys, they preferentially use old-growth forest. In contrast, we predict that between-site differences would not occur in redtail monkeys, because they prefer young successional forest, like that covering much of the disturbed site.

## Materials and methods

### Study site

**Figure 1:**
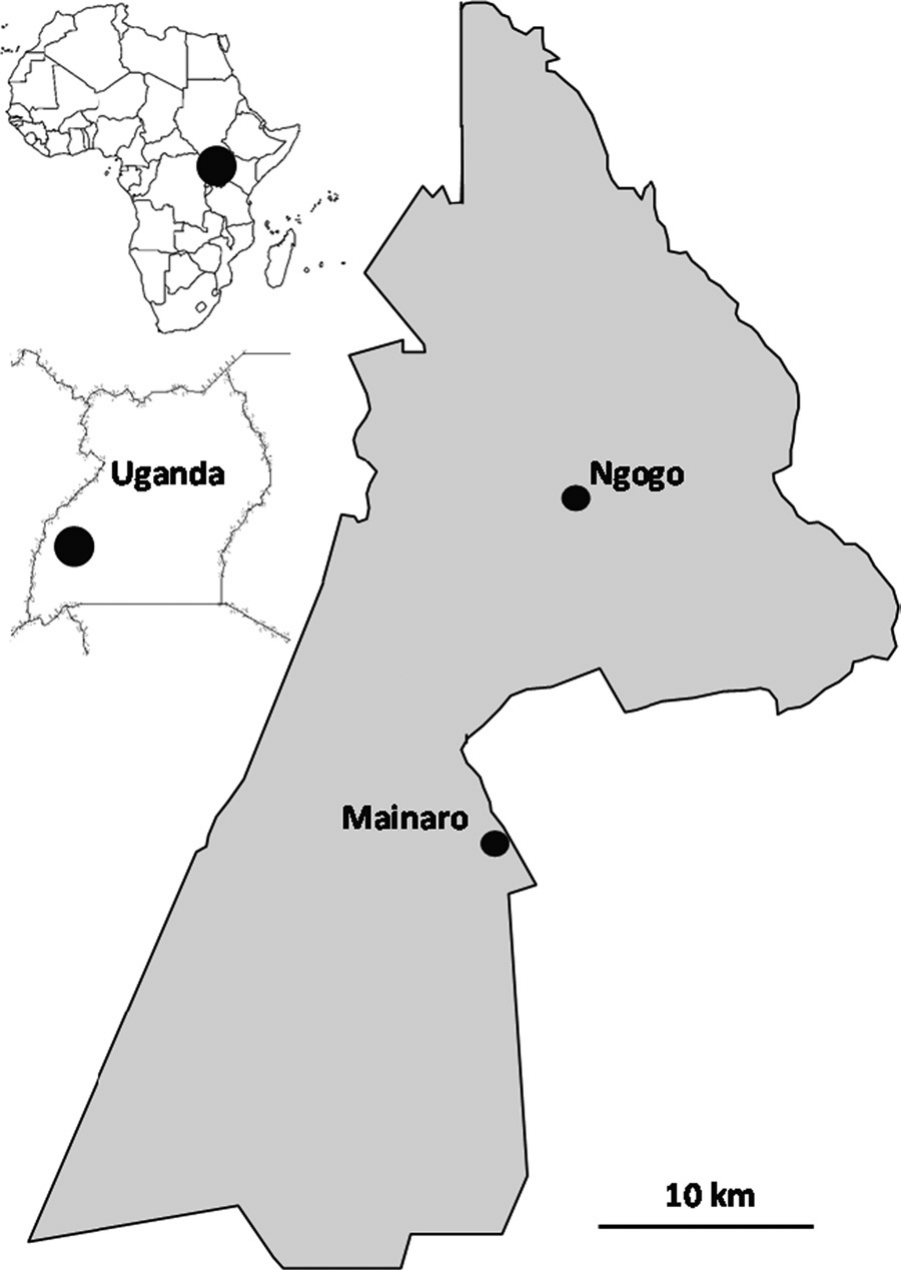
Map of Kibale National Park, Uganda.

Kibale National Park, Uganda (766 km^2^) is a moist, evergreen medium-altitude forest with a mosaic of old-growth forest, swamp, grassland and regenerating forest of varying ages (Struhsaker, 1997). Mainaro, on the southeastern edge of the park (Fig. 1), experienced severe encroachment by local farmers between the 1960s and the early 1990s despite Kibale's protected status (Van Orsdol, 1986; Aronsen, 2010). Replanting of endemic tree species was initiated in 1994, and the site now has a mosaic of old-growth forest patches interspersed with patches of previously disturbed vegetation at various stages of succession and a large area of replanted, young forest (Verweij and Emmer, 1998; Mucunguzi *et al*., 2007). Ngogo, in the centre of Kibale (Fig. 1), was not subjected to commercial logging while Kibale was a forest reserve (Struhsaker, 1997), nor did it experience recent conversion of forest to farmland. It contains large areas of old-growth forest plus areas of regenerating forest, swamp forest and grassland (Lwanga *et al*., 2000). Human presence is more conspicuous at Mainaro because it is on the edge of the park and immediately accessible via public road. Ngogo, at the centre of the park, is accessible only by a long, difficult dirt road and footpaths. Average diameter at breast height (dbh) for trees >10 cm dbh is similar at both sites [for Mainaro, 24.2 ± 16.31 cm (SD), *n* = 3447 trees, G. P. Aronsen and S. Teelen, unpublished data; and for Ngogo, 25.0 ± 24.0 cm, *n* = 2600 trees, C. A. Chapman, unpublished data]. However, Ngogo has more large trees (>50 cm dbh), reflecting its lack of recent human disturbance (Kolmogorov–Smirnov *Z* = 2.02, *P* = 0.001; G. P. Aronsen and S. Teelen, unpublished data; C. A. Chapman, unpublished data).

### Study taxa

Red colobus monkeys are colobines whose diet comprises mostly young leaves and petioles but also includes fruits and mature leaves from a variety of species (Struhsaker, 1975; Chapman and Chapman, 1999). They use a variety of habitat types and occupy some forest fragments outside the park (Lwanga, 2006), but they form relatively smaller groups and have higher parasite loads in forest fragments than within the park (Gillespie *et al*., 2003; Chapman *et al*., 2006). Red colobus at Ngogo are under severe predation pressure from the large chimpanzee community there; hunting offtakes have been as much as 188 monkeys within 7 months (Teelen, 2008). As a result, the local red colobus population has declined steeply in the last decade (Teelen, 2008; Lwanga *et al*., 2011; Watts and Amsler, 2013). Red colobus abundance is higher at Mainaro than at Ngogo; although limited data indicate a slightly higher mean group size at Ngogo (34–45 individuals per group for four groups; Teelen, 2007a,b, 2008) than at Mainaro (mean = 30 individuals per group; Chapman and Chapman, 2000), census data give much higher group abundance counts at Mainaro (mean = 0.29 groups/km; G. P. Aronsen and S. Teelen, unpublished data) than at Ngogo (mean = of 0.06 groups/km; Lwanga *et al*., 2011).

Redtail monkeys, which are common at both sites, are smaller than mangabeys and red colobus. They use a broad mix of vegetation types, including old-growth forest, but apparently prefer young, regenerating forest (Lambert, 1995; Sheppard, 1999; Baranga, 2004). Redtails in unprotected forest fragments also have higher parasite loads than those within the park (Gillespie *et al*., 2005), but they use areas where even colobines are uncommon (Chapman *et al*., 2003), indicating better ability to persist in suboptimal habitats. Redtail abundance may be slightly higher at Ngogo than at Mainaro; the mean number of groups encountered per kilometre during censuses there was 0.70 in 2007 (Lwanga *et al*., 2011), whereas the mean for Mainaro was 0.57 during 2007–2010 (G. P. Aronsen and S. Teelen, unpublished data). Published group size estimates are slightly higher for Ngogo (*n* = 32 individuals; Mitani *et al*., 2001) than for Mainaro (*n* = 25; Chapman and Chapman, 2000; Mitani *et al*., 2001), although the variation in group size is considerable (Struhsaker and Leland, 1988; Windfelder and Lwanga, 2004; Lwanga *et al*., 2011).

### Collection of urine and analysis of urinary cortisol

Animal encounter and urine collection followed the methods of Jaimez *et al*. (2012). Between June and August 2010, G.P.A. and field assistants walked along existing forest transects to find primate groups; on encountering one group, they estimated its size and followed it until they lost it or until the end of the day. In Kibale, redtail day ranges average about 1 km (Lambert, 1999); red colobus day ranges average ∼570 m (Isbell, 1983). Redtails sampled at Ngogo were habituated, but those at Mainaro were not, and red colobus were poorly habituated at both sites. To avoid generating additional stress via human presence, we attempted to avoid resampling the same group on consecutive days by spacing out collection locations by distances >1 km. This method was deployed successfully by Jaimez *et al.* (2012). In related species, urinary cortisol values reflect circulating concentrations 4–8 h in the past (Whitten *et al*., 1998), thereby reducing the potential impact of observational contact with researchers.

The study period occurred during what is typically a dry season, but note that neither energy availability nor reproduction shows strong seasonal variation in red colobus or redtails (Struhsaker, 1997). At each site, two different groups were sampled from each species (four groups from each site; eight groups in total). While following groups, we used plastic pipettes to collect urine from vegetation at the ground layer immediately after we saw an animal void. We immediately placed the urine into 1.5 ml snap tubes that were labelled with sample identification numbers and information on individual sex and age (if known), date and time of day. To minimize the risk of sample cross-contamination, urine was collected only when fresh and when it was clear that multiple individuals had not urinated in the same area. The GPS location was noted for each sample. No samples were collected on days when it rained. Collections were made over 15 day periods at each site. Monkeys at Mainaro were assigned to the disturbed forest (DF) group category, while those at Ngogo were assigned to the undisturbed forest (UF) group category. No monkeys are individually tagged or identifiable at these sites. We made every effort to avoid repeated sampling of single individuals by systematically moving through the group's spatial distribution and collecting samples during feeding, travel and resting. We did not resample individuals in an area where a team member had already walked through. We sampled across age and sex classes and pooled data for analysis. There is a risk of repeated sampling when studying any primate group, but our use of multiple groups and attempts to move exhaustively across the group over the course of a day provided our best effort to limit pseudoreplication.

Field handling and storage followed previously deployed and validated field protocols (Knott, 1997; Jaimez *et al*., 2012). At camp, 100–200 μl of urine (depending on amount collected) from each sample was pipetted onto an Atago PAL-10S Portable Digital Clinical Refractometer to record specific gravity to supplement creatinine measurements to correct for urine concentration. The refractometer was cleaned between samples. Each urine sample was then transferred onto Whatman filter paper. Each piece of filter paper was placed on aluminum foil and labelled with a sample identification number and with information on the sex and age of the individual (when known), date and time of day. Between 100 and 200 μl of urine was evenly pipetted onto the filter paper, in duplicate when the volume was sufficient. To protect samples against mould contamination, each piece of filter paper was immediately placed into a small ziplock bag that was in turn placed on aluminum foil resting over a layer of silica. These ziplock bags were placed in the shade and left undisturbed for 2 days (Campbell, 1994; Shideler *et al*., 1995). Once dried, samples were wrapped in foil, labelled with the sample number and placed in plastic slide sheets for storage and transport back to the Yale University Reproductive Ecology Laboratory.

Urinary cortisol was assayed as follows. Elution of the filter paper was conducted by insertion into labelled 16 mm × 100 mm borosilicate tubes. Five millilitres of 100% methanol was added to each test tube, and tubes were sealed with parafilm and refrigerated overnight. The following morning, the filter paper was squeezed against the side of the tube using sterilized forceps. Sample tubes were dried under compressed air or nitrogen, reconstituted with 1 ml of distilled water, vortexed for 2 min and sealed with parafilm until they were assayed (Knott, 2005; Marshall and Hohmann, 2005; Dittami *et al*., 2008). Creatinine values were also assessed via Jaffe's reaction (Taussky, 1954).

Creatinine correction was calculated by dividing the cortisol value by the creatinine value. Immediately after creatinine assays were completed, samples were assayed in duplicate using an unmodified high-sensitivity salivary cortisol enzyme immunoassay suitable for detecting the range of reconstituted urine hormone values (catalogue 1-3102; Salimetrics, State College, PA, USA). This assay has previously been deployed successfully in non-human primates (Jaimez *et al.*, 2012). Coefficients of variation for internal high- and low-quality control were 0.45 and 5.16%. Blanks read below detection levels. Samples that were excluded either had concentrations below detection limits of the assay or had insufficient volume.

### Statistical analysis

Results showed heteroscedastic distribution, so we logarithmically transformed raw data. Runs tests and residual plotting confirmed normality, with all results having a *P*-value > 0.05. Associations between cortisol and collection times were determined using standard linear regression. We used Student's unpaired *t*-tests to assess differences in mean urinary cortisol values. Statistical analyses were conducted using Prism 6.02 for Windows (GraphPad, Inc., San Diego, CA, USA). The value of α was set at 0.05.

This research adhered to the legal requirements of Uganda and to the American Society of Primatologists Principles for the Ethical treatment of Nonhuman Primates. The Yale University Institutional Animal Care and Use Committee issued a waiver for the research because of its strictly non-invasive methods.

## Results

**Figure 2:**
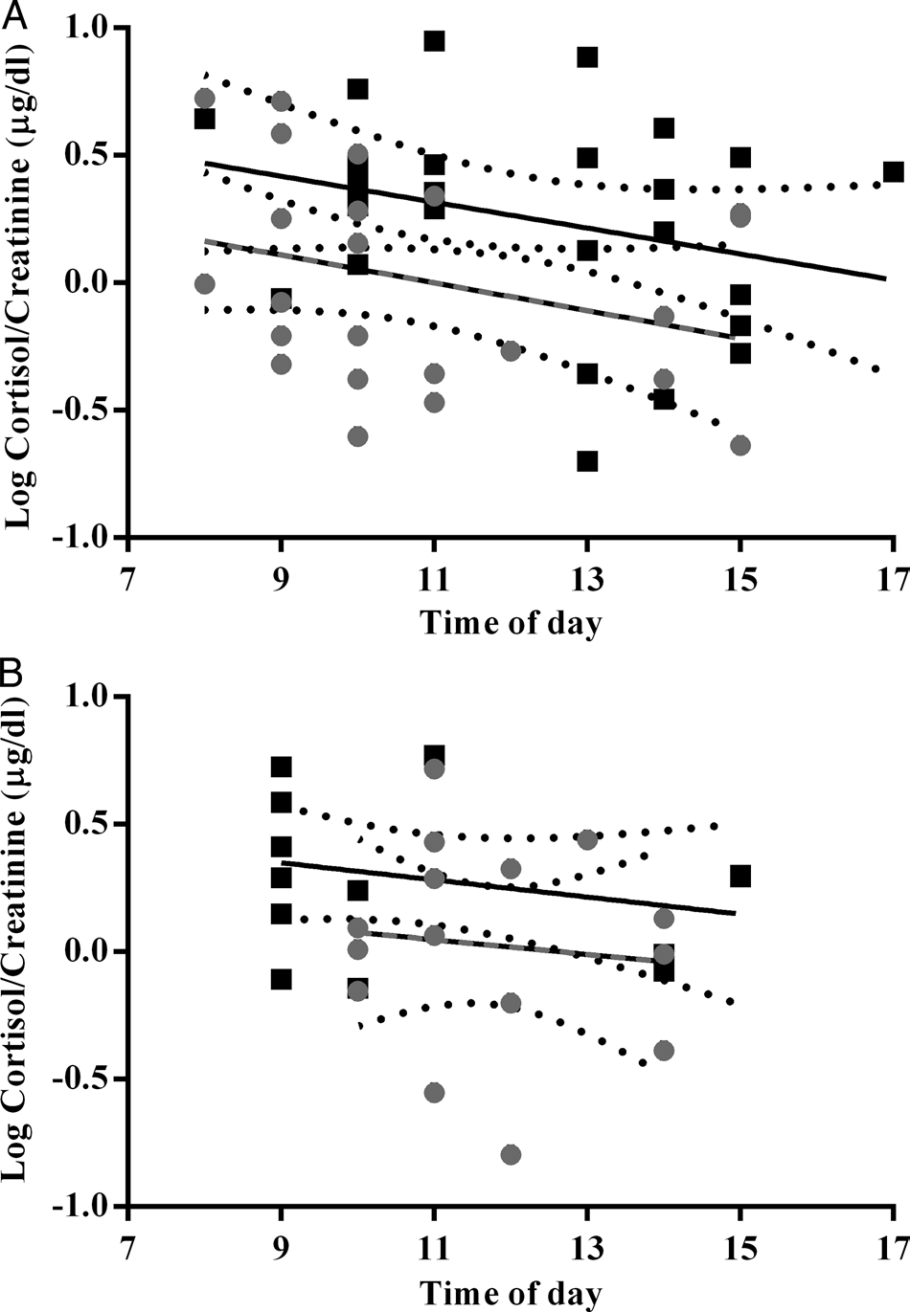
Daily variation in cortisol in redtail (left) and red colobus monkeys (right). Mainaro data are grey circles and dashed lines; Ngogo data are black squares and solid lines. The 95% confidence intervals are plotted.

**Figure 3:**
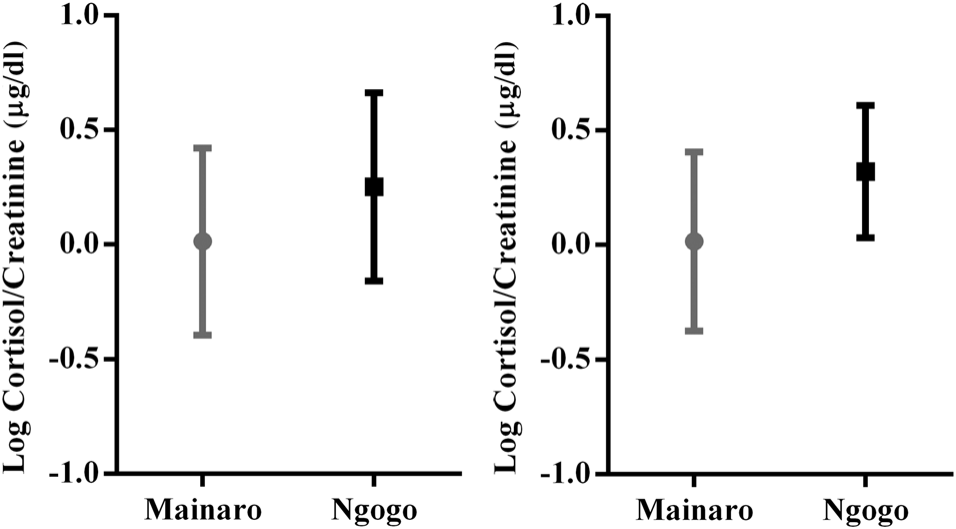
Logarithmically transformed cortisol concentrations for redtail (left) and red colobus monkeys (right). Means and standard deviations are shown. Mainaro (disturbed forest) is grey, and Ngogo (undisturbed forest) is black. Intersite variation is significant for both species (for redtails, *t* = 2.063, d.f. = 48, *P* = 0.045; and for red colobus, *t* = 2.536, d.f. = 28, *P* = 0.017).

We analysed 50 redtail samples (Mainaro, *n* = 26; and Ngogo, *n* = 24) and 30 red colobus samples (Mainaro, *n* = 17; and Ngogo, *n* = 13). Redtails and red colobus at both sites followed the expected circadian pattern of decreasing cortisol from morning to evening. For redtails, cortisol concentrations did not decline significantly in relationship to time of day at either site (Mainaro, β = −0.05, *r*^2^ = 0.09, *P* = 0.16; and Ngogo, β = −0.05, *r*^2^ = 0.08, *P* = 0.16; Fig. 2), but slopes for the two sites differed significantly (*F*_1,47_ = 7.26, *P* = 0.01). Diurnal variation in cortisol concentrations was not significant for red colobus at either site, nor did the slopes differ significantly (Mainaro, β = −0.03, *r*^2^ = 0.01, *P* = 0.71; and Ngogo, *β* = −0.03, *r*^2^ = 0.07, *P* = 0.34; slope comparison, *F*_1,26_ = 2.89, *P* = 0.10, Fig. 2). Contrary to our predictions, both red colobus and redtails had significantly higher cortisol concentrations at Ngogo than at Mainaro (redtails, *t*_48_ = 2.063, *P* = 0.045; and red colobus, *t*_28_ = 2.536, *P* = 0.017; Fig. 3).

## Discussion

Cortisol can serve as an indicator of physiological stress, and habitat disturbance can lead to increases in cortisol secretion. The hypothalamic–pituitary–adrenal feedback response is conservative across primates and largely consistent across the family Cercopithecidae (Coe *et al*., 1992). Our previous research showed that mangabeys had elevated cortisol at Mainaro relative to Ngogo, suggesting physiological stress due to habitat disturbance (Jaimez *et al*., 2012). In northern Kibale, mangabeys and red colobus in disturbed habitats show longer travel duration, lower body mass and (for red colobus) higher faecal cortisol concentrations relative to conspecifics in less disturbed areas (Olupot *et al*., 1994; Olupot, 2000; Gillespie *et al*., 2005; Chapman *et al*., 2006, 2007).

Given these results for mangabeys, we predicted similar cortisol elevations for Mainaro red colobus, but no difference between Mainaro and Ngogo for redtails. Instead, both species showed higher cortisol concentrations at Ngogo, the less disturbed of our two study sites. Groups at both sites also showed the expected diurnal decrease in cortisol concentrations, but the decrease was non-significant, suggesting physiological stress as the reason for the between-site differences. We therefore need an explanation for why these results differ from those reported earlier for mangabeys. Factors that may contribute independently or in combination to cortisol variation are described below.

### Environmental stressors

Plasma cortisol concentrations are influenced by environmental perturbations and subsequent changes to food and/or activity levels (Rhynes and Ewing, 1973; Follenius *et al.*, 1982; Martínez-Mota *et al*., 2007; Beehner and McCann, 2008; Foerster *et al*., 2012; Rangel-Negrín *et al*., 2014). Fragmented habitat structure may allow for increased insolation (and therefore ambient temperature) and increased travel time, but these variables are expected to affect Mainaro (disturbed) populations more than Ngogo (undisturbed). The study was conducted during the dry season at Kibale, but temperature and rainfall data for the study period were not markedly different from previous years (J. S. Lwanga, unpublished data).

### Intragroup competition

Interindividual agonism influences primate cortisol concentrations. Factors such as male–male competition, female dominance, resource access, reproductive status and increased group size can all lead to increased social conflict (Alberts *et al*., 1992; Strier *et al*., 1999; Creel, 2001; Muller and Wrangham, 2004; Fichtel *et al*., 2007; Arlet *et al*., 2009; Mendonça-Furtado *et al*., 2014). During our study period, group sizes were consistent with previously reported data (Chapman and Chapman, 2000; Struhsaker, 1975, 1980). Ngogo redtail group fission (and associated agonism) has been described (Windfelder and Lwanga, 2004), and thus, could be a source of intersite cortisol variation. The red colobus group distribution was relatively small at Ngogo during the study period (see Predation section below), making this explanation less viable. It is possible that we sampled Ngogo groups during a period of social instability, which could be tested via more long-term sampling of specific groups.

### Intergroup competition

Spatiotemporal variation in food availability can also lead to increased encounter rates and conflict between groups or species, thus altering primate cortisol concentrations (Pride, 2005; Schoof and Jack, 2013). While Ngogo has an old-growth forest, access to preferred fruiting trees/resources could lead to higher competition and associated increases in cortisol concentrations. During the study period, no interspecific/intergroup aggression or encounters were observed, and the fruits eaten by each species are neither high demand nor uncommon. At Mainaro, both redtails and red colobus fed on *Chrysophyllum albidum* and *Uvariopsis congensis* fruits most frequently. These resources are neither rare nor uncommon within Kibale. At Ngogo, redtails fed from these tree species, while red colobus were feeding most heavily on *Ptyerygota mildbraedii* fruits. This tree species is rare at Mainaro, but very common at Ngogo.

### Phytohormone contamination

The influence of exogenous sources on endocrine function has been described (Colborn *et al*., 1993; Tyler *et al*., 1998; Hotchkiss *et al*., 2008), but many aspects remain unclear (Retana-Márquez *et al.*, 2012; Zhabinskii *et al*., 2014). Research on Ugandan red colobus has shown that consumption of *Milettia dura* can have an effect on cortisol concentrations due to phytoestrogen concentrations (Wasserman *et al*., 2012a,b). This specific plant species was not eaten during the study period, and we are unaware of phytohormone studies of the plant species consumed during our study. This is an additional confounding factor, requiring both sampling of plants consumed by primates and analysis of these species' pharmacokinetic influence on endocrine function (Rode *et al*., 2006; Lu *et al*., 2011; Rothman *et al*., 2012; Wasserman *et al*., 2013b).

### Predation

Effects of predation pressure on cortisol concentrations are well documented in other animals (Tilbrook *et al*., 2000; Engh *et al*., 2006; Arlet and Isbell, 2009; Sheriff *et al*., 2011; Wang *et al*., 2011; Soares *et al*., 2012; Michelena *et al*., 2012; Cooke *et al*., 2013a; Fischer *et al*., 2014; but see Baker *et al*., 2013). Variation in predation rates may explain the cortisol concentration variation between Mainaro and Ngogo, especially for red colobus. Red colobus are the preferred prey of chimpanzees wherever the two species are sympatric (Boesch, 1994; Stanford, 1995; Watts and Mitani, 2002). The Ngogo chimpanzee community is the largest yet recorded and exerts unusually high predation pressure on the local red colobus population. While Ngogo chimpanzees hunt all diurnal primate species, effort and offtake is comparatively low for those other than red colobus. This is especially so for redtails and mangabeys, the most abundant primate species there (Lwanga *et al*., 2011). Predation by Ngogo chimpanzees has strongly affected red colobus demography and distribution (Mitani and Watts, 1999; Watts and Mitani, 2002; Pobiner *et al*., 2007; Teelen, 2008; Watts and Amsler, 2013). Census data show a steady decline of red colobus density within the chimpanzees' home range, with encounter rates for red colobus declining steeply since 1998, almost certainly because of predation by chimpanzees (Mitani *et al*., 2010; Lwanga *et al*., 2011; Watts and Amsler, 2013). Chimpanzees sometimes wait under or stalk red colobus groups for more than an hour before hunting them or leaving and sometimes attack the same group repeatedly over several hours (Watts and Mitani, 2002; D. Watts, personal observation). Besides their much lower frequency, hunts of redtails have much shorter durations (Watts and Mitani, 2002). By implication, exposure to chimpanzees should have much greater physiological effects on red colobus than on redtails or mangabeys, and these effects could easily outweigh any direct effects of variation in energy availability. Following a chimpanzee predation event, Kanyawara red colobus faecal samples (*n* = 6) showed elevated cortisol concentrations 2–5 days afterwards (Wasserman *et al*., 2013a). Given this, the pattern of elevated urinary cortisol in Ngogo red colobus may be associated with an unobserved chimpanzee predation event, constant vigilance for chimpanzee presence and/or variation in urinary/faecal cortisol concentrations following predation events (Bahr *et al*., 2000; Voellmy *et al*., 2014).

Crowned hawk-eagles (*Stephanoaetus coronatus*) are also major primate predators in Kibale (Skorupa, 1989; Struhsaker and Leakey, 1990), and redtails are their most common prey at Ngogo (Mitani *et al*., 2001; Sanders *et al*., 2003). Polyspecific associations between redtails and red colobus are more frequent at Ngogo than at Kanyawara, and redtails appear to initiate and maintain associations to limit eagle predation (Teelen, 2007a). Habitat disturbance strongly restricts crowned hawk-eagle distribution in the Kibale region, where the species occurs only inside the park (Seavy and Apodaca, 2002; Sekercioglu, 2002). How much its distribution within Kibale varies is unknown. Crowned hawk-eagle home range size is estimated at 10 km^2^, which implies that few pairs could use the Mainaro study area given its narrow east–west width (Fig. 1). Crowned hawk-eagles and other raptors may have higher densities or higher predation success at Ngogo than at Mainaro; if so, this could explain why redtail cortisol concentrations were lower at Mainaro.

Our previous study described higher concentrations of cortisol in mangabeys at Mainaro than at Ngogo, the inverse of the patterns seen in redtails and red colobus (Jaimez *et al*., 2012). Why does this contrast exist? Available data indicate that successful predation of mangabeys by chimpanzees and crowned hawk-eagles is relatively low (Ham, 1994; Waser, 1985; Watts and Mitani, 2002; D. P. Watts, unpublished data). Adult male mangabeys often chase crowned hawk-eagles; they show elevated faecal cortisol concentrations for days afterwards (Arlet and Isbell, 2009). At Ngogo, mangabeys usually flee from hunting attempts by chimpanzees, and, as for redtails, average hunt duration is short (Watts and Mitani, 2002). Consequently, chimpanzee predation attempts probably have limited physiological effects, and any such effects are likely to be outweighed by those of variation in food availability and variation in foraging effort.

Our preliminary results illustrate the complexity of associating endocrine variation with single variables such as habitat fragmentation. The increased cortisol concentrations recorded in Ngogo redtails and red colobus relative to their Mainaro conspecifics may be associated with one or several variables, such as social group instability, fruit and leaf phytochemistry and/or predation pressure. The downstream effects of increased cortisol concentrations on individual fitness are under debate (Sapolsky, 1985; Rabin *et al*., 1988; Goymann, 2012; Liu *et al.*, 2012; Boonstra, 2013a,b; Crespi *et al*., 2013; Dantzer *et al*., 2014). If increased cortisol has a detrimental impact on aspects of senescence, immune function and fertility, the Ngogo red colobus population, already under severe predation pressure, may dwindle further due to the lethal combination of high predation loss and severe psychological/physiological stress. For Ngogo redtails, increased cortisol concentrations may be an indicator of impending social group fission, hierarchy instability or other stressful events. At Mainaro, which is currently undergoing restoration, the relatively lower cortisol concentrations suggest that this habitat currently provides a suitable environment for redtail and red colobus monkeys. The Mainaro mangabeys, with their relatively higher requirements for mature, undisturbed forests, may still be struggling with environmental stressors.

Our study provides insights on the evaluation of primate condition and concerns within a national park, and the results are useful for conservation biology and park/site management protocols. The present results indicate that the Mainaro restoration efforts are providing suitable habitat for some, but not all, primate species, and continued efforts to bring Mainaro back into a more old-growth state will enhance and improve primate diversity. The results also indicate that primate population declines (red colobus) may result from biogenic (intra- or interspecific factors) rather than anthropogenic causes. Future studies at Kibale and for other primate species must combine physiology, socioecology, habitat structure, ecological community and nutritional data collection. Monitoring these variables will provide a more nuanced and cohesive evaluation of glucocorticoid responses in wild primates and provide measures of individual/community health and potential stressors.

## Funding

We received funding support from the Yale Institute for Biospheric Studies Program in Reproductive Ecology, the L.S.B. Leakey Foundation, the Great Ape Trust of Iowa and the Yale University Department of Anthropology.
